# Diffuse glioma with *FGFR3*::*TACC3* gene fusion and prominent calcification: A case report

**DOI:** 10.1016/j.radcr.2025.04.034

**Published:** 2025-05-03

**Authors:** Masaoki Kusunoki, Osamu Togao, Koji Yamashita, Kazufumi Kikuchi, Daisuke Kuga, Yutaka Fujioka, Shun Akamine, Kousei Ishigami

**Affiliations:** a Department of Clinical Radiology, Graduate School of Medical Sciences, Kyushu University, Fukuoka, Japan; b Department of Molecular Imaging and Diagnosis, Graduate School of Medical Sciences, Kyushu University, Fukuoka, Japan; c Department of Neurosurgery, Graduate School of Medical Sciences, Kyushu University, Fukuoka, Japan; d Department of Anatomic Pathology, Graduate School of Medical Sciences, Kyushu University, Fukuoka, Japan

**Keywords:** Brain tumor, Diffuse glioma, Glioblastoma, FGFR3, TACC3, Calcification

## Abstract

Diffuse gliomas with fibroblast growth factor receptor 3 (*FGFR3*) and transforming acidic coiled-coil containing protein 3 (*TACC3*) gene fusion represent a distinct molecular subtype of isocitrate dehydrogenase (IDH)-wildtype gliomas characterized by unique histopathological features. Although microcalcifications have frequently been reported in histopathological studies, their prevalence and diagnostic significance on radiological imaging remain unclear. We report a case of a 67-year-old woman who presented with a 1-year history of weakness in the left lower limb. Head CT revealed coarse and irregular calcifications in the deep white matter of the right frontal and parietal lobes. At the same time, MRI demonstrated a diffuse gliomatosis cerebri-like growth pattern with infiltration across the corpus callosum and contrast enhancement in distant areas. Histopathological examination confirmed glioblastoma, IDH-wildtype, and subsequent genetic testing revealed *FGFR3*::*TACC3* fusion and amplification of *FGFR3* gene. This case highlights the potential radiological characteristics of diffuse gliomas with *FGFR3*::*TACC3* fusion, particularly the presence of coarse calcifications, that may serve as notable imaging features of this tumor. Further research is required to determine whether calcification is a characteristic of this glioma subtype.

## Introduction

In the 2021 World Health Organization (WHO) classification of central nervous system tumors, adult-type diffuse gliomas are broadly categorized into astrocytomas, Isocitrate Dehydrogenase (IDH)-mutant, oligodendrogliomas, IDH-mutant and 1p/19q-codeleted, and glioblastomas, IDH-wildtype [1]. Within the IDH-wildtype subset, diffuse gliomas with *FGFR3*::*TACC3* gene fusion represent a distinct molecular subtype first identified by Singh et al. in 2012 [2]. This fusion involves fibroblast growth factor receptor 3 (FGFR3) and transforming acidic coiled-coil containing protein 3 (TACC3), leading to the constitutive activation of oncogenic signaling pathways that promote tumor growth and progression [3,4]. *FGFR3*::*TACC3* fusion has been identified in approximately 4% of diffuse gliomas, IDH-wildtype, highlighting the distinct molecular and pathological characteristics that distinguish it from other glioma subtypes [2,[5], [6], [7], [8], [9], [10]]. Patients with *FGFR3*::*TACC3* fusion-positive glioblastomas were reported to be similar in age or slightly older at diagnosis compared to those with fusion-negative glioblastomas, with variable reports regarding sex predominance [[6], [7], [8]].

*FGFR3*::*TACC3* fusion-positive gliomas demonstrate a relatively favorable prognosis, with better outcomes than *FGFR3*::*TACC3*-negative glioblastomas, although still poorer than those of IDH-mutant gliomas [[6], [7], [8]]. The presence of this fusion introduces potential therapeutic options, including FGFR-targeted therapies, highlighting the importance of accurate molecular characterization in guiding treatment decisions [5,11,12]. Notably, the *FGFR3*::*TACC3* fusion plays a role in tumorigenesis in various cancers, including bladder [13], lung [14], and uterine cervical malignancies [15], underscoring its broader oncogenic significance.

These tumors may present with distinctive imaging features, such as prominent calcifications, that can aid in the differential diagnosis. However, the radiological imaging characteristics of *FGFR3*::*TACC3* fusion-positive gliomas have not yet been fully elucidated, posing challenges in distinguishing them from other diffuse gliomas.

Here, we report a case of diffuse glioma with *FGFR3*::*TACC3* fusion, characterized by striking calcifications and a diffuse infiltrative growth pattern consistent with glioblastoma, IDH-wildtype. This case highlights the importance of integrating radiological, pathological, and molecular findings to improve diagnostic accuracy and inform clinical management.

## Case presentation

A 67-year-old woman presented to a local hospital with a 1-year history of weakness in the left lower limb. Imaging examinations performed at the hospital suggested the presence of a brain tumor, prompting a referral to the neurosurgery outpatient department. The patient had no notable medical or family history, and laboratory tests revealed no significant abnormalities. At the initial visit to our hospital, she reported subjective weakness in the left lower limb; however, neurological examination did not reveal any apparent motor deficits or other neurological abnormalities. Manual muscle testing (MMT) showed full strength (5/5) in both lower limbs, and deep tendon reflexes and muscle tone were normal. Further imaging studies, including computed tomography (CT) and magnetic resonance imaging (MRI), were performed at our radiology department.

**Fig. 1:**
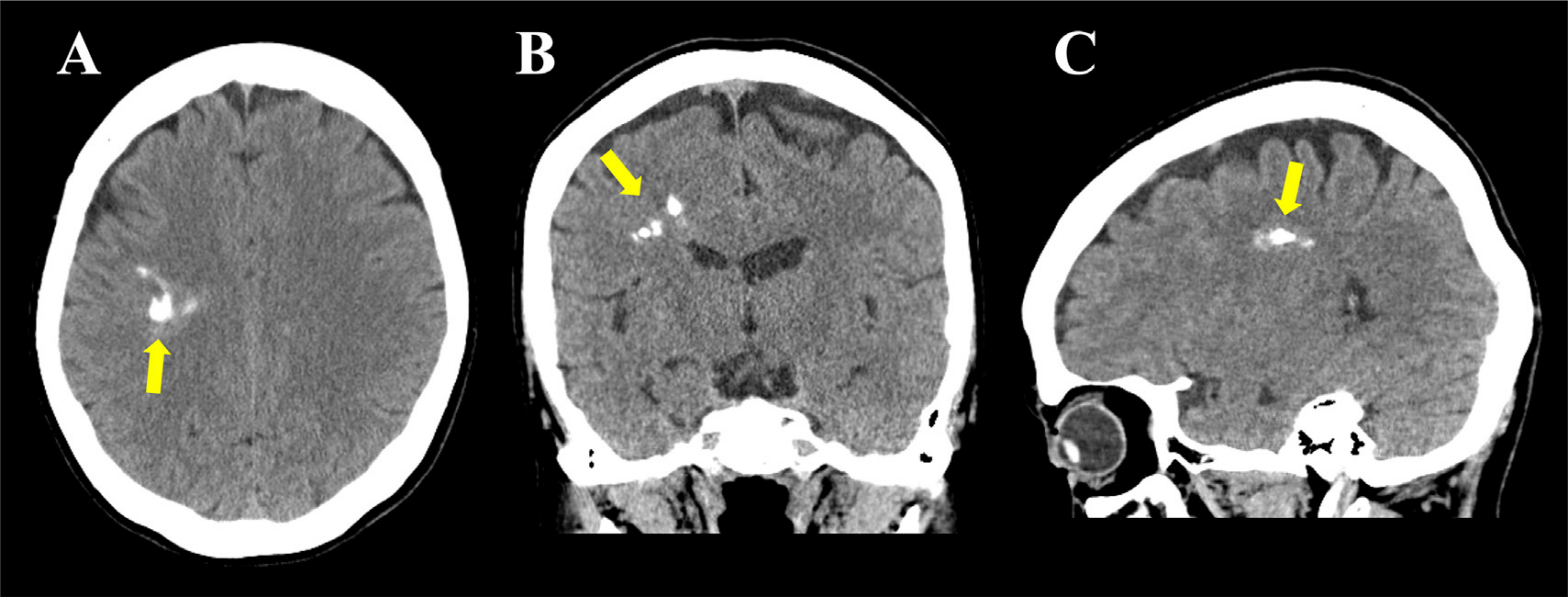
Head CT findings. Noncontrast CT images of the head in the axial (A), coronal (B), and sagittal (C) views show irregular and heterogeneous coarse calcifications (arrows) in the deep white matter of the right frontal and parietal lobes.

Noncontrast head CT (Fig. 1A–C) revealed multiple irregular and heterogeneous calcifications in the deep white matter from the right frontal to the parietal lobes, predominantly involving the white matter of the medial precentral and postcentral gyri.

**Fig. 2:**
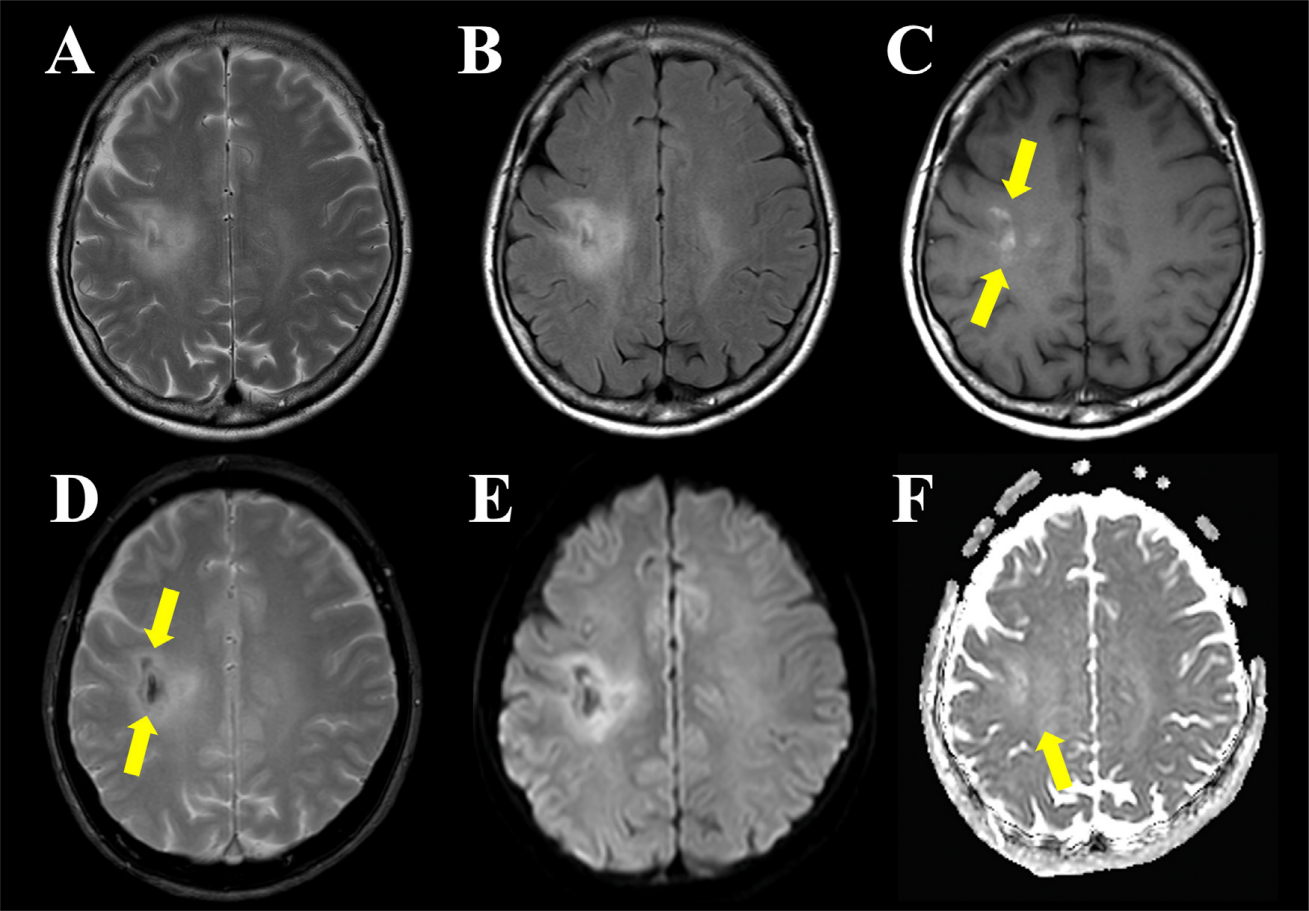
Brain MRI findings. Brain MRI with T2-weighted (A) and FLAIR (B) imaging shows an ill-defined area of high signal intensity extending across the right frontal and parietal lobes. In addition, faint hyperintensity on T1-weighted imaging (C, arrow) and hypointensity on T2*-weighted imaging (D, arrow) in the deep white matter of the right frontal and parietal lobes suggest the presence of calcifications. Diffusion-weighted imaging (E) shows hyperintensity in the deep white matter of the right frontal and parietal lobes, while the apparent diffusion coefficient (ADC) values are comparable to those of the contralateral normal white matter (F, a mean ADC value of 0.83 × 10⁻³ mm²/s in the area indicated by the arrow).

**Fig. 3:**
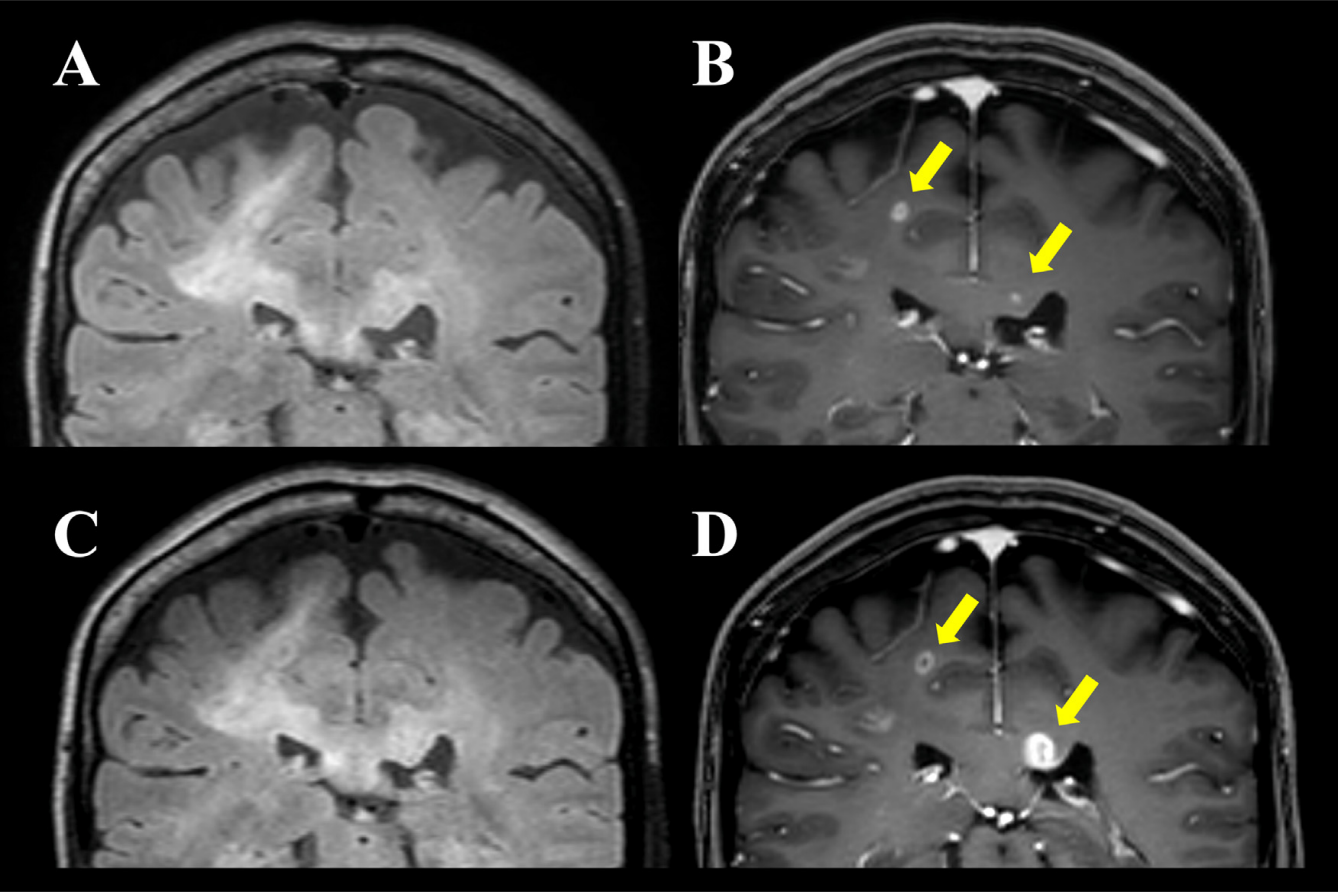
Brain MRI findings. Brain MRI in coronal view at initial evaluation at our hospital shows that the ill-defined high signal intensity area on FLAIR imaging (A) extends to the left side of the corpus callosum, with enhancement observed in the medial right precentral and postcentral gyri as well as the left corpus callosum on contrast-enhanced T1-weighted imaging (B, arrows). Follow-up MRI 2 months later shows no change in the ill-defined high signal intensity area on FLAIR imaging (C); however, enlargement of the enhancing lesions and ring-like enhancement are noted on contrast-enhanced T1-weighted imaging (D, arrows).

Brain MRI revealed an ill-defined hyperintense area on T2-weighted images (Fig. 2A) and T2-fluid attenuated inversion recovery (FLAIR) imaging (Fig. 2B) extending from the deep white matter of the right frontal lobe to the parietal lobes, crossing the corpus callosum to the contralateral white matter (Fig. 3A). Correspondingly, high signal intensity on T1-weighted imaging (Fig. 2C) and low signal intensity on T2*-weighted imaging (Fig. 2D) were observed, indicating calcification in the deep white matter of the right frontal and parietal regions. On diffusion-weighted imaging (DWI; b = 1000 s/mm^2^ in 3 orthogonal directions with a slice thickness of 5 mm and an interslice gap of 1 mm), hyperintensities were identified in the lesions within the right frontal to parietal lobes and corpus callosum (Fig. 2E). The apparent diffusion coefficient (ADC) value of 0.83 × 10⁻³ mm²/s was comparable to or slightly higher than that of normal white matter (Fig. 2E). Contrast-enhanced T1-weighted imaging revealed punctate enhancement in the white matter of the medial aspects of the right precentral and postcentral gyri, as well as the left side of the corpus callosum (Fig. 3B).

Two months later, follow-up MRI revealed no significant changes in the ill-defined T2 prolongation (Fig. 3C), but enlargement of the enhancing lesions in the white matter of the medial right precentral and postcentral gyri and the left side of the corpus callosum formed a ring-like shape (Fig. 3D). Glioblastoma, IDH wildtype, was strongly suspected, and a stereotactic brain tumor biopsy was performed targeting the lesion on the left side of the corpus callosum for diagnostic confirmation.

**Fig. 4:**
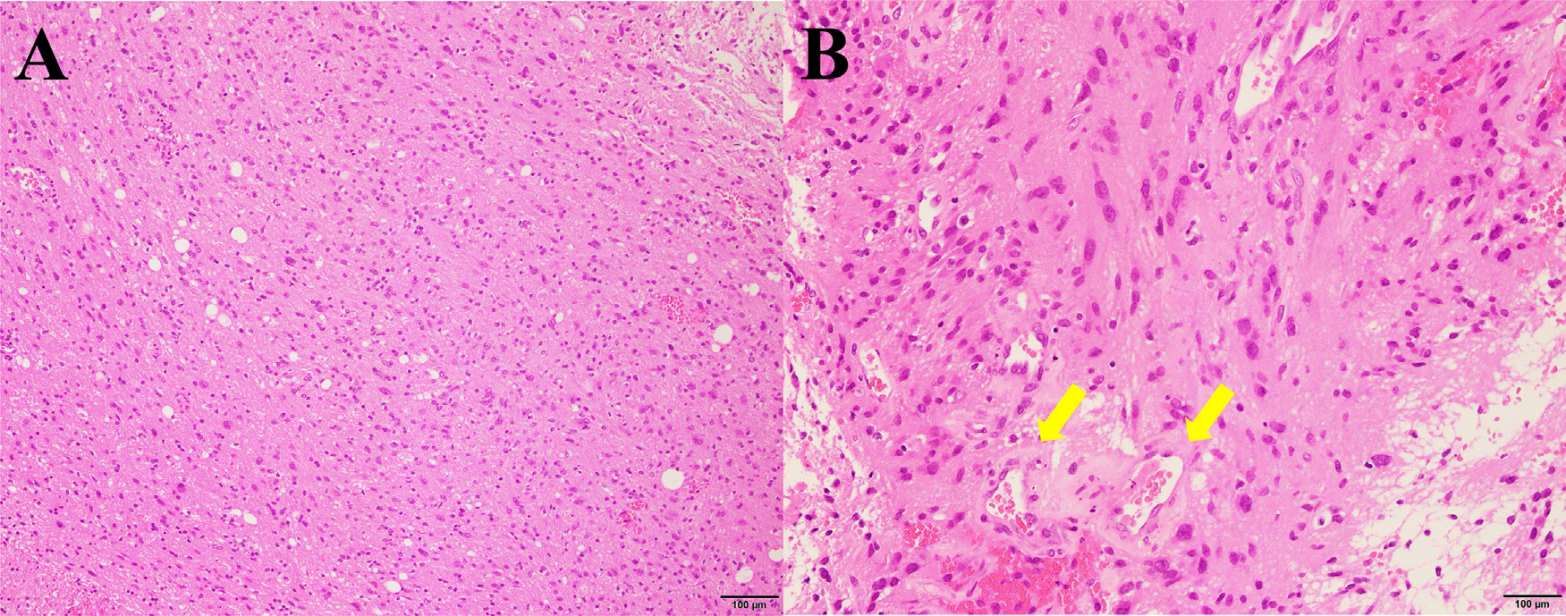
Histopathological findings. Biopsy specimen obtained from the left side of the corpus callosum (hematoxylin and eosin staining) shows diffuse proliferation of glioma cells at low magnification (A) and microvascular proliferation at high magnification (B, arrows).

Histopathological and molecular analyses of the biopsied lesion confirmed the diagnosis of glioblastoma, IDH-wildtype. Histopathological examination revealed diffuse proliferation of anaplastic glioma cells with enlarged, pleomorphic nuclei (Fig. 4A). Focal mitotic figures were observed (5/10 high-power fields), and microvascular proliferation was suspected (Fig. 4B); however, palisading necrosis was not evident. Additionally, no microcalcifications were observed in the biopsy specimens, possibly due to the location of the sampling site.

Immunohistochemical staining revealed that the glioma cells were focally positive for p16 and p53, negative for IDH1 R132H, and retained ATRX and MTAP. The MIB-1 labeling index was 10%. Molecular analysis revealed IDH1/2-wildtype, 1p/19q noncodeletion, *BRAF* wildtype, *TERT* promoter mutation, *EGFR* gene copy number gain, and *MGMT* promoter methylation. Based on these findings, a diagnosis of glioblastoma, IDH-wildtype was made according to the 2021 updated 5th WHO Classification of Tumors of the Central Nervous System (WHO CNS5) criteria.

Subsequently, the patient underwent chemoradiotherapy with temozolomide and maintenance chemotherapy with bevacizumab and/or temozolomide. With the patient’s consent, a cancer gene panel test was performed, which identified *FGFR3*::*TACC3* fusion (F17*; T4) and *FGFR3* amplification (copy number = 10). Follow-up MRI examinations performed over > 18 months showed no worsening of symptoms or lesion progression.

## Discussion

Diffuse gliomas with *FGFR3*::*TACC3* gene fusion were first identified as distinct disease entities by Singh et al. in 2012 [2]. This fusion-type glioma is recognized for its unique molecular and pathological characteristics that differentiate it from other gliomas [9]. *FGFR3*::*TACC3* fusion occurs in approximately 4% of glioblastomas, IDH-wildtype and a similar proportion of diffuse gliomas, IDH-wildtype with lower-grade histology [2,[5], [6], [7], [8],^10^]. Patients with *FGFR3*::*TACC3* fusion-positive glioblastomas were reported to be similar in age or slightly older at diagnosis, with no significant differences in laterality or anatomical localization compared to those with fusion-negative glioblastomas [[6], [7], [8]]. The *FGFR3*::*TACC3* fusion results from the juxtaposition of *FGFR3*, encoding a receptor tyrosine kinase involved in cell proliferation and growth, and *TACC3*, encoding a microtubule-associated protein implicated in mitotic spindle stabilization [[16], [17], [18]]. These genes, located 48 kb apart on chromosome 4p16, fuse to drive oncogenesis by activating multiple signaling pathways, including MAPK, PI3K/AKT, and those involved in metabolic reprogramming through mitochondrial biogenesis and oxidative phosphorylation [2,4,19,20]. Molecularly, these tumors are negative for IDH1/2 mutations, 1p/19q codeletions, *H3* mutations, and BRAF p.V600E mutations, with rare EGFR amplification (0-5.6%) [[5], [6], [7], [8]]. Immunohistochemical staining for *FGFR3* demonstrated high sensitivity and specificity for detecting the *FGFR*3::*TACC*3 fusion protein, with reported values of 100% and 92% [21], and 93% and 95% [22], respectively. Given its high diagnostic accuracy, FGFR3 immunostaining may serve as a useful screening tool prior to genetic testing. In addition, recent studies have reported that RT-qPCR screening of formalin-fixed, paraffin-embedded samples achieves high sensitivity (100%) and specificity (83.3%), further supporting its potential for clinical application [23]. Diffuse gliomas with *FGFR3*::*TACC3* fusion have an intermediate prognosis, with poorer survival outcomes than IDH-mutant gliomas but superior outcomes compared to *FGFR3*::*TACC3*-negative glioblastomas [7]. In a recent study, the median overall survival of *FGFR3*::*TACC3* fusion-positive glioblastomas was reported to be 31.1 months, significantly longer than that of fusion-negative glioblastomas (19.9 months), with the fusion serving as an independent predictor of better outcome [7]. These tumors may also respond to FGFR inhibitors [5,11,12], emphasizing the critical role of molecular testing in guiding targeted therapies.

Histopathologically, diffuse gliomas with *FGFR3*::*TACC3* fusion frequently exhibit microcalcifications, nuclear palisading, and curvilinear capillary proliferation, which have been reported to be significantly more prominent than those in *FGFR3*::*TACC3*-negative glioblastomas [8,9,21,24]. Additionally, perivascular pseudorosette formation and oligodendroglial-like cells have been observed, although their diagnostic relevance remains unclear [9,21]. In the present case, the biopsy specimen obtained from the left side of the corpus callosum did not exhibit these characteristic features. Biopsies of the right frontal and parietal white matter, where calcifications were more prominent, were avoided because of the risk of motor neuron injury. It is plausible that specimens from this region exhibit more typical histological features. This suggests that histopathological differences may exist between rapidly growing, contrast-enhancing lesions and areas with prominent calcification. Molecular analysis confirmed IDH1/2-wildtype status, absence of 1p/19q codeletion, and presence of a *TERT* promoter mutation, consistent with glioblastoma IDH-wildtype per the WHO CNS5 criteria [1]. These molecular features are consistent with those reported previously, and no unique findings were observed in our case.

MRI depicts the histopathological features of glioblastoma, including tumor infiltration, angiogenesis, and necrosis, which are characterized by ill-defined, diffusely infiltrative mass lesions with heterogeneous high signal intensity on T2-weighted and FLAIR imaging. Contrast enhancement varies and may present as a solid, ring-like, nodular, or patchy pattern, possibly in multiple locations. Gliomatosis cerebri-like growth patterns are frequently observed in glioblastomas, particularly in IDH-wildtype cases, reflecting their highly infiltrative nature [25]. Previous radiological imaging studies of *FGFR3*::*TACC3* fusion-positive gliomas have reported a tendency for these tumors to localize to the cortex or subcortical white matter, particularly in the frontal or temporal lobes, with relatively well-circumscribed margins in a subset of cases based on MRI [7,8]. Contrast enhancement patterns are variable, ranging from minimal to ring-like enhancement, and tumor borders may appear either well-circumscribed or poorly defined [7,8,26,27]. Taken together, these findings suggest a wide spectrum of radiological appearances in *FGFR3*::*TACC3* fusion-positive gliomas. Given that glioblastomas in general exhibit considerable radiological diversity, the imaging features in our case would fall within the range described in previous reports.

However, the prevalence of radiologically detectable calcifications on CT and/or MRI in *FGFR3*::*TACC3-*positive diffuse gliomas remains unclear. On MRI, the calcium-induced T1 shortening, which depends on its concentration within the tissue, can result in hyperintensity on T1-weighted images [28,29]. These imaging findings may also reflect microcalcifications frequently reported in the histopathology of *FGFR3*::*TACC3* fusion-positive gliomas [27]. Although this correlates with the frequent microcalcifications observed on histopathological examinations, comprehensive imaging-based analyses remain limited. If calcification is a characteristic feature, radiological imaging can significantly enhance diagnostic accuracy. Further studies are needed to compare the frequency and distribution of calcifications in *FGFR3*::*TACC3* fusion-positive and -negative IDH-wildtype glioblastomas.

Considering the presence of calcifications, differential diagnoses include *FGFR3*::*TACC3* fusion-negative glioblastoma, oligodendroglioma, ependymoma, ganglioglioma, and polymorphous low-grade neuroepithelial tumors of the young (PLNTY). Among these, oligodendrogliomas, IDH-mutant, and 1p/19q-codeleted are typically located in the frontal lobe with cortical-subcortical involvement, commonly occur in younger adults, and may exhibit a cortical high-flow sign on arterial spin labeling (ASL), aiding in their noninvasive differentiation from other diffuse gliomas [30]. Supratentorial ependymomas in adults can mimic diffuse gliomas with *FGFR3*::*TACC3* fusion when displaying infiltrative growth. In contrast, gangliogliomas and PLNTYs, classified as WHO CNS5 grade 1 tumors, have low malignant potential, lack diffuse infiltration, and primarily affect individuals from childhood to young adulthood.

In the present case, the gliomatosis cerebri-like growth pattern, characterized by diffuse infiltration across the corpus callosum with contrast enhancement in distant areas, was highly suggestive of glioblastoma, IDH-wildtype. Notably, coarse calcifications in the deep white matter have emerged as a distinctive radiological feature that may aid in differentiating diffuse gliomas with *FGFR3*::*TACC3* fusion from fusion-negative glioblastomas. While previous reports have suggested an association between *FGFR3*::*TACC3* fusion and microcalcifications on histopathological examination, their prominence in radiological imaging underscores the need for further research to clarify their diagnostic and clinical relevance. As molecular characterization increasingly guides therapeutic decision-making, recognizing such radiological features may enhance diagnostic accuracy and support the early identification of patients who could benefit from FGFR-targeted therapies.

## Conclusion

Diffuse gliomas with *FGFR3*::*TACC3* fusion constitute a distinct molecular subtype with potentially unique radiological features, particularly prominent calcifications with an infiltrative tumor growth pattern suggestive of glioblastoma, IDH-wildtype, which may serve as a useful diagnostic marker. Accurate identification of this entity is essential, given its intermediate prognosis and potential responsiveness to FGFR-targeted therapies. Further studies are warranted to elucidate the clinical significance of calcifications and refine the diagnostic and therapeutic approaches for affected patients.

## Patient consent

The patient provided written informed consent for the publication of this case study.
